# The Labeled Square Root Cubature Information GM-PHD Approach for Multi Extended Targets Tracking

**DOI:** 10.3390/s26020367

**Published:** 2026-01-06

**Authors:** Zhe Liu, Siyu Zhang, Zhiliang Yang, Xiqiang Qu, Jianping An

**Affiliations:** 1 School of Information and Communication Engineering, North University of China, Taiyuan 030051, China; 2 School of Cyberspace Science and Technology, Beijing Institute of Technology, Beijing 100081, China

**Keywords:** Gaussian mixture, square root cubature information filter, ET-GM-PHD, labeled GM components, trajectory constructing

## Abstract

For modern radars with high resolutions, an extended target may generate more than one observations. The conventional point target-based tracking method can hardly be applied in such scenarios. Recently, the ET-GM-PHD approach has been presented for tracking these extended targets. The performance of such an approach has been influenced by the following disadvantages. First, it has been formulated under the linear Gaussian assumptions. When targets move with nonlinear models, the tracking performance may be rapidly decreased. Second, it neglects the time associations of the estimated states at different time steps, which makes it very challenging to manage targets for the radar systems. In this paper, we present a labeled ET-GM-PHD approach based on the square root cubature information filter (SRCIF) to solve such problems. To be more specific, we, first, utilize the SCRIF for predicting and updating the GM components of the ET-GM-PHD approach. For decreasing the computational cost, a candidate observation extracting method has been put forward in the GM component updating step. Thus, the ET-GM-PHD approach can be adopted to track extended targets with nonlinear motions. Second, a label-based trajectory constructing method has been proposed. By assigning the GM components with different labels before the GM component predicting step, we can obtain the estimated states with different labels. On this basis, the associations between the estimated states and trajectories can be modeled based on these labels. Thus, we can obtain the states and trajectories of multi extended targets simultaneously. The simulation results prove the effectiveness of our approach.

## 1. Introduction

### 1.1. Background

With the rapid development of the electronic technique, modern radars with high resolutions have been widely used in military and civilian fields [1]. Using these high-resolution radars, we can obtain more than one observation generated by a target. Such a target has been named the extended target [2,3,4,5]. In practice, it is rather important to estimate the various number and states based on observations from high-resolution radars. Commonly, it has been named as the multi extended target tracking. The conventional target tracking methods have been derived under the standard point target assumption [6,7,8,9,10]. Under such an assumption, the state of each target has been associated with no more than one observation. Thus, the conventional target tracking approaches can hardly be applied in extended target tracking scenarios. Therefore, extended target tracking becomes a rather challenging problem in many domains, such as unmanned aerial vehicles, radar systems, and remote sensing [11,12,13].

To solve the extended target tracking problems, researchers have proposed many approaches. Feldmann et al. [14] derived that the extension of the extended target can be modeled by the random symmetric positive definite matrix. However, it is rather hard to quantify the estimations theoretically. Tuncer et al. [4] apply the variational Bayes (VB) technique to describe the time-varying orientation of the extended targets. Thus, it can simultaneously achieve the orientation and states of an extended target. Jiao et al. [15] used the variational Bayesian inference in distributed sensor networks. By deriving the distributed VB observations, the proposed approach can be employed to implement the Bayesian estimation for the extended target. Yang et al. [16] derived the copula function to describe the dependencies of observations. It can be applied to track extended targets with dependent observations. A Gaussian process model of observations under the probabilistic multi-hypothesis tracker (PMHT) framework has been presented in [17]. It can process the extended and point-like target seamlessly. However, the associations between states and observations are rather difficult to achieve. Granström et al. [18] proposed the Poisson multi-Bernoulli mixture (PMBM) approach for tracking multi extended targets. In such an approach, the detected and undetected targets have been modeled by the Poisson point process and multi-Bernoulli mixture. Then, the Gaussian inverse Wishart gamma has been raised to solve the data association problems of the extended targets. Such an approach has been applied to track the coexisting point and extended targets in [19]. Xie et al. [20] integrated the multiple models into the PMBM filter. With the aid of such an implementation, the PMBM filter can accommodate multi extended target maneuvering problems. Although these MB-based approaches have high tracking performance, the computational complexity is more expensive than the probability hypothesis density (PHD)-based approaches. The probability hypothesis density (PHD) filter for multi extended target observation models has been derived in [21]. Such a filter is computationally intractable. By modeling the target extensions with random matrices, the PHD of the extended target can be approximated by the gamma Gaussian inverse Wishart distributions (namely, the GGIW-PHD approach) in [22]. As for this approach, the extended target has been assumed to have an ellipsoidal shape. Gong et al. [23] modeled the extended target with a varying number of sub-objects. Then, the GGIW-PHD approach can be employed to solve the non-ellipsoidal extended target tracking problems. However, the tracking performance was dependent on the number of sub-objects. Granstrom et al. [24] represented the PHD of extended target by a group of Gaussian mixture (GM) components. Using these components, the complex integrals of PHD equations can be substituted by the summation of the GM components. This approach has been named the ET-GM-PHD approach. Compared with the above random matrix-based filters, the ET-GM-PHD focuses on estimating the states of extended targets, and it has less computational complexity. Although the ET-GM-PHD approach has rather efficient computation complexity, it only considered the linear tracking scenarios. Yan et al. [25] use the Fuzzy C-Means (FCS) to cluster the observations of extended targets. Chen et al. [26] applied the cubature Kalman filter (CKF) into the ET-GM-PHD approach. It simply employs all observations to compute the posterior intensity. When the number of targets grows large, the computational complexity increases rapidly. For tracking 3D multi extended targets, Yang et al. [27] formulated the Gaussian process regression observation model. Then, it was integrated into the ET-GM-PHD framework. Thus, the kinematic state and shape of the targets can be simultaneously obtained. Addressing the observation partition problem, Qin et al. [28] presented a density-based clustering algorithm. Using this algorithm, observations of the multi extended targets can be partitioned efficiently. In order to obtain the target extension of the ET-GM-PHD approach, Zhang et al. [29] adopted the convolutional conditional neural process to estimate the shape function. However, it relies on the prior. Jiang et al. [30] derived the update equation of the PHD filer in target-dependent false alarms. It can be applied to track the individual target within a group. Cao et al. [31] presented the extended target trajectory probability hypothesis density (ETTPHD) filter for tracking the extended traffic target. By developing the interactive motion model for collision avoidance and lane constraint models, the ET-GM-PHD approach can be employed for tracking the extended traffic target. Nevertheless, it has been derived for the target neighboring and dense cases. The fuzzy adaptive resonance theory (ART) model has been presented for clustering the observations of extended targets in [32]. The estimating accuracy of this approach has greatly depended on the selection of vigilance values. Lin et al. [33] present the LPHD filter to improve the tracking performance of the standard particle PHD filter. The δ-generalized LPHD (δ-GLPHD) filter has been proposed in [34]. By incorporating the information of the LPHD filter into the pre-processing step, the tracking performance of the LPHD filter can be significantly improved. Mao et al. [35] derived the labeled PHD filtering approach. Using the SMC implementation, such an approach can be adopted to solve nonlinear non-Gaussian scenarios. However, it has been formulated under the standard point target assumption. In addition, it also neglects the influence of target missing. When the target is missing at a certain time step, the trajectory of the target may be interrupted.

### 1.2. Our Work and Contribution

Recently, the information filters have been widely used in multi-sensor fusion. In contrast to the conventional Kalman filters, the information filters are easier to be initialized and have lower computational load. Among these filters, the square root cubature information filter (SRCIF) can be applied to solve the state estimation problem with high dimensions. In addition, it has significant estimation performance in nonlinear target tracking scenarios. In this paper, we improve the tracking performance of the ET-GM-PHD approach in nonlinear scenarios using the SCIF method. Here, we name our approach the labeled square root cubature information ET-GM-PHD (LSRCI-ET-GM-PHD) approach. To be more specific, we first adopt the SRCIF method to predict and update the labeled GM components. To employ such a method in extended target tracking scenario, we derive a candidate observation extracting strategy in the update step of the SRCIF method. Thus, the updated GM components with labels can be achieved. Then, the state extracting method has been proposed to extract the states of targets from these labeled GM components. On this basis, we present a label-based trajectory constructing method. Using such a method, we can construct the trajectory of each target by the estimated states. Thus, the tracking performance of the proposed approach can be significantly improved. The main contributions of our approach have been listed as follows.

(1) We present a labeled ET-GM-PHD approach based on the SRCIF method. First, we use a group of GM components to describe the density of the extended target. These GM components have been assigned different labels. Then, these labeled GM components have been predicted by the SRCIF method. Since the SRCIF method has been put forward for tracking standard targets, it cannot be directly applied to update the GM components of the extended targets. To solve such a problem, we have raised a candidate observation extracting method. With such a method, we can obtain the observations of each partition. Then, we implement the updating step of the SRCIF method to evaluate the updated labeled GM components. Using the updated components, we can obtain the posterior densities of multi extended targets. Benefiting from the above implementations, the tracking performance of the proposed approach can be significantly improved.
(2) We present the state extracting method of our approach. Since we applied the labeled GM components for predicting and updating the density, GM components with the same label have a larger probability of being associated with the same target than the others. According to this, we first use the pruning method of the conventional ET-GM-PHD approach to discard the GM components with small weights. Thus, the number of GM components can be greatly decreased. Then, we derive the merging method to combine GM components with the same label. With the help of the preset threshold, these combined labeled components can be merged to extract the states of the multi extended targets.
(3) The label-based trajectory constructing method has been proposed for constructing the trajectories of multi extended targets. In multi extended target tracking scenarios, affected by clutters, target detection loss, and death, the trajectory of a target may be broken into several pieces. To avoid such problems, we first divide the targets into four sets, such as, the survival, death, undetected, and unconfirmed sets. These sets can be used to describe the cases, such as target birth, survival, detection loss, and so on. Obviously, states with the same label at different time steps belong to the same target. Thus, we can assign the estimated states into the survival sets based on the label. When the labels of estimated states are not in the survival set, we present the label assignment strategy based on the gating method and trajectories. With such a strategy, the trajectories of extended targets can be steadily constructed.

The rest of this paper is organized as follows: We overview the GM-PHD approach for extended targets in Section 2. Section 3 proposes our LSRCI-ET-GM-PHD approach. Simulation results are demonstrated in Section 4, and Section 5 concludes this paper.

## 2. The GM-PHD Approach for Extended Target Tracking

In this section, we overview the PHD filter for the extended target in Section 2.1. Then, the GM implementation of the ET-PHD filter has been introduced in Section 2.2.

### 2.1. The PHD Filter for Extended Target Tracking

Before introducing the basic idea of the extended PHD filter, we assume that there are $M_{k}$ extended targets at time step *k*. We denote the states of these targets by $X_{k}$,

(1) $$X_{k}=\left\{x_{1,k},x_{2,k},\ldots ,x_{M_{k},k}\right\},$$

where $x_{i,k}$ is the state of the *i*-th target,

(2) $$x_{i,k}=\left(p_{x,k}^{i},p_{y,k}^{i},v_{x,k}^{i},v_{y,k}^{i}\right).$$

$\left(p_{x,k}^{i},p_{y,k}^{i}\right)$ denotes the position of the current target, and $\left(v_{x,k}^{i},v_{y,k}^{i}\right)$ is the corresponding velocity.

In multi target tracking scenarios, due to the target appearing, disappearing, and maneuvering, $x_{i,k}$ and $M_{k}$ in (1) are time varying. Notice that the state $x_{i,k}$ in $X_{k}$ has no ordering, and the number $M_{k}$ is finite. Therefore, $X_{k}$ can be considered as the random finite set (RFS).

Let $Z_{k}$ denote the observation set at time step *k*, $z_{i,k}$ be the *i*-th observation, and $z_{j,k}=\left(p_{x,k}^{j},p_{y,k}^{j}\right)$ ($j=1,2,\ldots ,N_{k}$, $N_{k}$ is the number of observations). As mentioned before, each extended target may generate more than one observations. That is to say, for each $x_{i,k}$, there may exist more than one corresponding observations. These observations are always partitioned into several subsets (namely, the cells [3]). As for the *p*-th partition, we have

(3) $$Z_{p}=\left\{Z_{1,k}^{p},Z_{2,k}^{p},\ldots ,Z_{c,k}^{p}\right\},$$

where $Z_{c,k}^{p}$ denotes the *c*-th cell at the current partition.

Obviously, for each pair of $Z_{j,k}^{p}$ and $Z_{j,k}^{p}$ in the (3), we have

(4) $$Z_{i,k}^{p}\cup Z_{j,k}^{p}=Ø,\forall Z_{i,k},Z_{j,k}\subset Z_{k}.$$

For simplicity, we use $Z_{c,k}$ to denote $Z_{c,k}^{p}$ in this section. $Z_{c,k}$ can be represented by

(5) $$Z_{c,k}=\left\{z_{1,k}^{c},z_{2,k}^{c},\ldots ,z_{N_{c},k}^{c}\right\},$$

and $N_{c,k}$ denotes the number of observations in the current cell.

From the (3) and (5), we can find that the observation set of multi extended targets can be depicted by several cells. Each cell represents one possible target. Thus, the likelihood of the conventional point target cannot be directly applied. To solve such a problem, Gilholm [36] adopted the Poisson model to describe the likelihood of the single extended target by

(6) $$l_{x}\left(x_{i,k}\right)=e^{-\beta (x_{i,k})}\prod_{z\in Z_{c,k}}\beta \left(x_{i,k}\right)\phi_{z}\left(x_{i,k}\right).$$

Here, $\beta (x_{k})$ represents the number of observations generated by $x_{k}$, and $\phi_{z}\left(\cdot \right)$ denotes the likelihood of the single target in case of a single observation.

Considering the clutters and detection possibility, (10) can be rewritten by

(7) $$l_{z}\left(x_{i,k}\right)=e^{-\beta (x_{i,k})}p_{D}\left(x_{i,k}\right)\prod_{z\in Z_{j,k}}\frac{\beta \left(x_{i,k}\right)\phi_{z}\left(x_{i,k}\right)}{\lambda_{k}c_{k}\left(z_{k}\right)}$$

where $\lambda_{k}$ and $c_{k}\left(\cdot \right)$ denote the number and spatial distribution of clutters at the current time step.

With the likelihood of the single extended target in (7), Mahler [21] derived the extended-target PHD filter (EPHD) to track extended targets. Similar to the PHD filter in standard target tracking, the EPHD filter consists of two steps, the predicting and updating steps. We introduce the EPHD filter as follows.

Let $D_{k-1|k-1}\left(\cdot \right)$ be the density of a single target at time step k−1. The prediction step can be modeled by (8). In (8), we define $p_{s}\left(\cdot \right)$ as the survival probability, $\varphi_{k|k-1}\left(\cdot \right)$ as the transition density, and γ(·) as the intensity of birth targets.

(8) $$D_{k|k-1}\left(x_{k}|Z_{1:k-1}\right)=\underset{\mathrm{Survival}\mathrm{intensity}}{\underset{︸}{\int_{X}p_{s}\left(x_{k}\right)\varphi_{k|k-1}\left(x_{k}|x\right)D_{k-1|k-1}\left(x|Z_{1:k-1}\right)dx}}+\underset{\mathrm{Birth}\mathrm{intensity}}{\underset{︸}{\gamma_{k}\left(x_{k}\right)}}.$$

Then, we implement the updated step of the EPHD filter by (9).

(9) $$D_{k|k}\left(x_{k}\right)=L_{z}\left(x_{k}\right)D_{k|k-1}\left(x_{k}|Z_{1:k-1}\right),$$

where $L_{z}\left(x_{k}\right)$ can be considered as the pseudolikelihood function,

(10) $$L_{z}\left(x_{k}\right)=1-\left(1-e^{\beta (x_{k})}\right)p_{D}\left(x_{k}\right)+e^{\beta (x_{k})}p_{D}\left(x_{k}\right)\sum_{℘∠Z_{k}}\omega_{℘}\sum_{W∠℘}\frac{\beta {\left(x_{k}\right)}^{\left|\beta (x_{k})\right|}}{d_{W}}\cdot \prod_{z\in W}\frac{\phi (x_{k})}{\lambda_{k}c_{k}\left(z\right)},$$

(11) $$\omega_{℘}=\frac{\prod_{W\in ℘}d_{W}}{\sum_{℘∠Z_{k}}\prod_{W}d_{W}}.$$

(12) $$d_{W}=\delta_{|W|,1}+\int D_{k|k-1}\left(x|Z_{1:k-1}\right)e^{\beta (x)}\beta {\left(x\right)}^{\left|\beta (x)\right|}p_{D}\left(x\right)\prod_{z\in W}\frac{\phi (x_{k})}{\lambda_{k}c_{k}\left(z\right)}dx,$$

In (11) to (12), $℘∠Z_{k}$ denotes that *℘* partitions the observation set $Z_{k}$ into nonempty cells W. $\omega_{y}$ denotes the weight of the current partition, and $\delta_{i,j}$ is the Kronecker delta. Since the associations of the observations are usually unknown beforehand, we can only apply the possible partitions in (10). For example, let $Z=\{z_{1},z_{2},z_{3}\}$; all of the possible partitions can be clustered by ${℘}_{1}=\left\{\left\{z_{1},z_{2},z_{3}\right\}\right\}$, ${℘}_{2}=\left\{\left\{z_{1}\right\},\left\{z_{2},z_{3}\right\}\right\}$, ${℘}_{3}=\left\{\left\{z_{1},z_{2}\right\},\left\{z_{3}\right\}\right\}$, ${℘}_{4}=\left\{\left\{z_{1},z_{3}\right\},\left\{z_{2}\right\}\right\}$, and ${℘}_{5}=\left\{\left\{z_{1}\right\},\left\{z_{2}\right\},\left\{z_{3}\right\}\right\}$. Obviously, when the number of observations is large, it may incur a high computational load on observation partitioning.

Equations (8)–(12) describe the recursion of the EPHD filter. However, there are no closed forms for these equations.

### 2.2. The GM-PHD Filter for Extended Target Tracking

In this section, we overview the ET-GM-PHD filter. The ET-GM-PHD filter can be seen as an implementation of the EPHD filter. Like the GM-PHD in standard target tracking, it also describes the density of a single target by a group of GM components. Then, these GM components have been applied in (8)–(12). With the help of these components, we can convert the integral operations into the summation of GM components. Thus, the EPHD filter can be tractable for computing the density of extended targets. The main details of the ET-GM-PHD filter have been provided as follows.

In order to implement the ET-GM-PHD filter, Granstrom [24] has made several assumptions.

(1) Both the dynamic and observation models of the extended targets are subject to linear Gaussian models, represented by
  (13) $$\begin{array}{ccc} \varphi_{k|k-1}\left(x_{k}|x_{k-1}\right) & ∼ & N(x;F_{k|k-1}x_{k-1},Q_{k-1}) \end{array}$$
  (14) $$\begin{array}{ccc} \phi_{k}\left(z_{k}|x_{k}\right) & ∼ & N(z_{k};H_{k}x_{k},R_{k})is \end{array}$$
  where N(:;m,P) denotes the Gaussian distribution, and m and P are the mean and covariance, respectively. $F_{k|k-1}$ is the transition matrix, and $H_{k}$ is the observation matrix.
(2) The possibilities of target survival and detection are state independent,
  (15) $$\begin{array}{ccc} p_{s}\left(x_{k}\right) & = & p_{s,k} \end{array}$$
  (16) $$\begin{array}{ccc} p_{D}\left(x_{k}\right) & = & p_{D,k} \end{array}$$
(3) The birth intensity is formulated by the GM components.
  (17) $$\gamma_{k}\left(x\right)=\sum_{i=1}^{J_{r,k}}w_{r,k}^{i}N\left(x;m_{r,k}^{i},P_{r,k}^{i}\right),$$
  where $w_{r,k}^{i}$ denotes the weight of the GM component, and $m_{r,k}^{i}$ and $P_{r,k}^{i}$ are the mean and covariance, respectively.

With above assumptions, Granstrom derives the GM forms of the EPHD filter. Such an implementation is named the ET-GM-PHD filter. Similar to the GM-PHD filter in standard target tracking, the predicted density in (8) can be rewritten by

(18) $$D_{k|k-1}\left(x\right)=v_{s,k|k-1}\left(x\right)+\gamma_{k}\left(x\right),$$

where $v_{s,k|k-1}\left(x\right)$ is the predicted density of the survival target,

(19) $$v_{s,k|k-1}\left(x\right)=p_{s,k}\sum_{j=1}^{J_{k-1}}w_{k-1}^{j}N\left(x;m_{k|k-1}^{j},P_{k|k-1}^{j}\right),$$

where $J_{k-1}$ is the number of the GM components at time step k−1, and $\left\{w_{k-1}^{j},m_{k|k-1}^{j},P_{k|k-1}^{j}\right\}$ represents the parameters of the predicted GM components.

Considering the observation set $Z_{k}$, we can express the updated density by

(20) $$D_{k|k}\left(x\right)=D_{k|k}^{ND}\left(x\right)+D_{k|k}^{D}\left(x\right).$$

In (20), $D_{k|k}^{ND}\left(x\right)$ and $D_{k|k}^{D}\left(x\right)$ denote the densities of the no-detection and detected target cases, respectively.

(21) $$\begin{array}{ccc} D_{k|k}^{ND}\left(x\right) & = & \sum_{j=1}^{J_{k|k-1}}w_{k|k}^{j}N\left(x;m_{k|k}^{j},P_{k|k}^{j}\right), \end{array}$$

(22) $$\begin{array}{ccc} w_{k|k}^{j} & = & \left(1-\left(1-e^{-\beta (x)}\right)\right)p_{D}\left(x\right)w_{k|k-1}^{j}, \end{array}$$

(23) $$\begin{array}{ccc} m_{k|k}^{j} & = & m_{k|k-1}^{j},P_{k|k}^{j}=P_{k|k-1}^{j}, \end{array}$$

(24) $$\begin{array}{ccc} D_{k|k}^{D}\left(x\right) & = & \sum_{j=1}^{J_{k|k-1}}w_{k|k}^{j}N\left(x;m_{k|k}^{j},P_{k|k}^{j}\right), \end{array}$$

(25) $$\begin{array}{ccc} w_{k|k}^{j} & = & \omega_{y}\frac{\Gamma^{j}p_{D}\left(x\right)}{d_{W}}\Phi_{W}^{j}w_{k|k-1}^{j}, \end{array}$$

(26) $$\begin{array}{ccc} \Gamma^{j} & = & e^{-\beta (x)}-\beta {\left(x\right)}^{\left|\beta (x)\right|}, \end{array}$$

(27) $$\begin{array}{ccc} \Phi_{W}^{j} & = & \phi_{W}^{j}\prod_{z\in W}\frac{1}{\lambda_{k}c_{k}\left(z\right)}. \end{array}$$

where $\phi^{j}\left(\cdot \right)$ is the likelihood of a single target.

Since the ET-GM-PHD filter has been derived under the linear Gaussian assumption, $\left\{m_{k|k}^{j},P_{k|k}^{j}\right\}$ for each GM component can be obtained by the Kalman filters. By implementing the above equations iteratively, the posterior densities of the multi extended targets can be obtained. However, in practice, subject to the target nonlinear maneuvering and observing, the linear Gaussian assumption may not be satisfied. Thus, the tracking performance of the ET-GM-PHD filter may be greatly declined.

## 3. The LSRCI-GM-PHD Algorithm for Extended Target Tracking

In this section, we put forward our LSRCI-ET-GM-PHD approach in detail. More specifically, we first present the labeled SRCIF-based ET-GM-PHD approach in Section 3.1. By predicting and updating the labeled GM components with the SRCIF and observation extracting methods, the posterior densities of the multi extended targets in nonlinear scenarios can be obtained. Second, a state estimating method has been derived in Section 3.2. With such a method, the states and numbers of extended targets of our LSRCI-ET-GM-PHD approach can be estimated. Third, we present a trajectory constructing method for the LSRCI-ET-GM-PHD approach in Section 3.3. Using such a strategy, we can construct the stable trajectory for each target.

### 3.1. The Labeled SRCIF-Based GM-PHD Method for Extended Target Tracking

In Section 2, the intensities $v_{s,k|k-1}$ and $D_{k|k}^{D}\left(x\right)$ in (19) and (24) are represented by a group of unlabeled GM components. These GM components can be obtained by computing their corresponding means and covariances. That is to say, GM components can be described by their corresponding means and covariances. Thus, we define the *i*-th labeled GM component at time *k* by $\left\{l^{i},m_{k}^{i},P_{k}^{i}\right\}$, where $l^{i}$ denotes the label of the *i*-th GM components, and $m_{k}^{i}$ and $P_{k}^{i}$ refer to the corresponding state and covariance. Notice that, $m_{k}^{i}$ and $P_{k}^{i}$ have been predicted and updated under the linear Gaussian assumption. For extending the ET-GM-PHD filter into the nonlinear target tracking scenarios, we tend to use the SRCIF method to evaluate $m_{k}^{i}$ and $P_{k}^{i}$. Although the SRCIF method has significant performance in nonlinear target tracking, it has been limited by the standard target assumption. To solve such a problem, we also present an observation extracting strategy for our approach. The main details of our approach are given in the following.

Suppose that the labels of the GM components do not change in the predicting and updating step in the SRCIF method. We can substitute $m_{k}^{i}$ and $P_{k}^{i}$ by $m_{k}$ and $P_{k}$ in this section for simplicity.

To introduce our approach, we rewrite the models of (13) and (14) as

(28) $$\begin{array}{ccc} \varphi_{k|k-1}\left(x_{k}|x_{k-1}\right) & ∼ & N(x;f\left(x_{k-1}\right),Q_{k-1}) \end{array}$$

(29) $$\begin{array}{ccc} \phi_{k}\left(z_{k}|x_{k}\right) & ∼ & N(z_{k};h\left(x_{k}\right),R_{k}) \end{array}$$

where both f(·) and h(·) are nonlinear functions, describing targets with nonlinear maneuvering and observing.

Equations (28) and (29) denote the nonlinear maneuvering and observing models, respectively. Under such models, the proposed method consists of the following three steps.

#### 3.1.1. Predict the State and Square Root Factor of the Covariance

In this step, we calculate $m_{k|k-1}$ and $S_{k|k-1}$ by $m_{k-1}$ and $P_{k-1}$, where $S_{k|k-1}$ is the square root factor of $P_{k|k-1}$ ($P_{k|k-1}=S_{k|k-1}S_{k|k-1}^{T}$). According to the SRCIF in [37], $m_{k|k-1}$ and $S_{k|k-1}$ are obtained by a group of cubature points. Using the obtained $m_{k|k-1}$ and $S_{k|k-1}$, the predicted density $v_{s,k|k-1}$ in (19) can be achieved.

For convenience, we introduce the cubature rule in [38] by

(30) $$I_{N}\left(c\right)=\int_{R^{n}}c\left(x\right)N\left(x;m,P\right)\approx \frac{1}{2n}\sum_{j=1}^{2n}c\left(m+\sqrt{P}\alpha_{j}\right)$$

where *n* is the dimension of m,

(31) $$\begin{array}{ccc} \alpha_{j} & = & \sqrt{n}{\left[1\right]}_{j} \end{array},j=1,2,3,\ldots ,2n$$

and ${\left[1\right]}_{j}$ is the *j*-th vector of the set

$$\left\{\left[\begin{array}{c} 1\\ 0\\ \vdots \\ 0 \end{array}\right],\ldots ,\left[\begin{array}{c} 0\\ \vdots \\ 0\\ 1 \end{array}\right],\left[\begin{array}{c} -1\\ 0\\ \vdots \\ 0 \end{array}\right],\left[\begin{array}{c} 0\\ \vdots \\ 0\\ -1 \end{array}\right]\right\}.$$

Let ${\left\{\chi_{k-1,j}\right\}}_{j=1}^{2n}$ be the cubature set. Using (30), we can represent the *j*-th cubature point at time step k−1 by

(32) $$\chi_{k-1,j}=\sqrt{P_{k-1}}\alpha_{j}+m_{k-1},$$

Then, these points can be propagated by

(33) $$\chi_{k-1,j}^{*}=f\left(\chi_{k-1,j},Q_{k-1}\right),$$

Using the above propagated points, the predicted state $m_{k|k-1}$ can be estimated by

(34) $$m_{k|k-1}=\frac{1}{2n}\sum_{j=1}^{2n}\chi_{k-1,j}^{*}$$

We calculate the square root factor of $P_{k|k-1}$ by

(35) $$S_{k|k-1}=\mathrm{Tria}(\left[C_{k|k-1,j},S_{Q,k-1}\right]),$$

where

(36) $$\begin{array}{ccc} C_{k|k-1,j}=\frac{1}{\sqrt{2d}} & [\chi_{k-1,1}^{*}-m_{k|k-1} & \ldots \\ & \chi_{k-1,2n}^{*}-m_{k|k-1}] & , \end{array}$$

Tria(·) denotes the QR decomposition, and $S_{Q,k-1}$ is the square root factor of $Q_{k-1}$.

Using (34) and (35), the predicted observation $z_{k|k-1}$ can be obtained by

(37) $$\begin{array}{ccc} z_{k|k-1} & = & \frac{1}{2n}\sum_{j=1}^{2n}\chi_{k|k-1,j}^{*}, \end{array}$$

where

(38) $$\chi_{k|k-1,j}^{*}=\varphi \left(\chi_{k|k-1,j}\right),$$

and

(39) $$\chi_{k|k-1,j}=S_{k|k-1}\alpha_{j}+m_{k|k-1}.$$

#### 3.1.2. Extract the Candidate Cells

In Section 2.2, all of the cells in each partition have been applied to compute the posterior density. When the number of observations is high, we may have a high number of possible partitions and cells. Directly using these cells and partitions may incur high computational complexity. To avoid such a problem, we, in this section, only use part of the cells to update the density. In multi target tracking scenarios, observations closed to the predicted observation have a high possibility to be generated by the same target. That is to say, cells closed to the predicted observation $z_{k|k-1}$ are more likely to be generated by the current target. Due to this, we describe the association between the cell $Z_{c,k}$ and predicted observation $z_{k|k-1}$ by

(40) $$\begin{array}{ccc} p(Z_{c,k}|z_{k|k-1}) & = & p(z_{1,k}^{c},z_{2,k}^{c},\ldots ,z_{|W_{c}|,k}^{c}|z_{k|k-1}),\\ & = & \prod_{j=1}^{|W_{c}|}p\left(z_{j,k}^{c}|z_{k|k-1}\right), \end{array}$$

where $Z_{c,k}$ is the *c*-th cell of the current partition, $z_{j,k}^{c}$ is the *j*-th observation of $Z_{c,k}$, and $|W_{c}|$ is the number of observations in $Z_{c,k}$. $z_{k|k-1}$ can be computed by the (37).

According to (29), $p(z_{j,k}|z_{k|k-1})$ can be represented by

(41) $$p\left(z_{j,k}^{c}|z_{k}\right)=N\left(z_{j,k}^{c};z_{k|k-1},R_{k}\right).$$

Substituting (41) into (40), we can rewrite (40) as follows,

(42) $$p\left(Z_{c,k}|z_{k|k-1}\right)=\prod_{j=1}^{|W_{c}|}N\left(z_{j,k}^{c};z_{k|k-1},R_{k}\right).$$

Then, we define the association possibility between $Z_{c,k}$ and $z_{k|k-1}$ by normalizing (42),

(43) $$p_{z}\left(Z_{c,k}\right)=\frac{p(Z_{c,k}|z_{k|k-1})}{\sum_{c=1}^{|}W|p\left(Z_{c,k}|z_{k|k-1}\right)},$$

where |W| denotes the number of cells in the current partition.

Obviously, when $Z_{g,k}$ is located nearby $z_{k|k-1}$, $p_{z}$ in (43) may have a higher value than the other cells. Thus, the candidate cell set can be extracted by

(44) $${\tilde{C}}_{k}=\left\{Z_{c,k}|p_{z}\left(Z_{c,k}\right)>T_{c}\right\},$$

where $T_{c}$ is the preset threshold.

According to (44), we extract the cells with high conditional probabilities. These extracted cells are adopted to update the states and covariance. Since cells with small probabilities have been excluded, the computational complexity can be saved.

#### 3.1.3. Update Predicted States and Covariances

In this step, we first use observations of the candidate cells to calculate the square root factor of the innovation covariance. Then, the information forms of the posterior state and square root factor of the covariance can be estimated by updating the predicted states and covariances.

According to [38], the innovation covariance can be computed by

(45) $$P_{ZZ,k|k-1}=\frac{1}{2n}\sum_{j=1}^{2n}\chi_{k|k-1,j}^{*}{\left(\chi_{k|k-1,j}^{*}\right)}^{T}-z_{k|k-1}z_{k|k-1}^{T}+R_{k}$$

To evaluate the posterior state and covariance, we convert $m_{k|k-1}$ and $S_{k|k-1}$ into their information forms by

(46) $$y_{k|k-1}=Y_{s,k|k-1}m_{k|k-1},$$

and

(47) $$Y_{s,k|k-1}={\left(S_{k|k-1}\right)}^{-1}.$$

where $y_{k|k-1}$ and $Y_{s,k|k-1}$ represent the information state and matrix, respectively.

Let $z_{j,k}$ be the *j*-th observation in the extracted cell set ${\tilde{C}}_{k}$, and *D* be the number of observations in ${\tilde{C}}_{k}$. Using (46) and (47), the SRCIF observation updating step can be described by

(48) $$\left[\begin{array}{ccccc} Y_{S,k|k-1} & Y_{1,M,k|k-1} & Y_{2,M,k|k-1} & \ldots & Y_{D,M,k|k-1}\\ y_{S,k|k-1}^{T} & z_{1,k}^{T}Y_{R,k} & z_{2,k}^{T}Y_{R,k} & \ldots & z_{D,k}^{T}Y_{R,k} \end{array}\right]\Theta =\left[\begin{array}{cc} Y_{S,k} & 0\\ y_{s,k} & ★ \end{array}\right],$$

where

(49) $$Y_{M,k|k-1}=Y_{S,k|k-1}^{T}P_{ZZ,k|k-1}^{T/2}Y_{R},$$

Θ is evaluated by the Householder reflections, and $Y_{R}$ is the square root factor of the information form of R.

By deriving (48), we can obtain $y_{s,k}$ and $Y_{S,k}$. Then, the posterior state $m_{k}$ and $P_{k}$ can be achieved by

(50) $$\begin{array}{cc} m_{k} & ={Y_{s,k}}^{-1}y_{k}, \end{array}$$

(51) $$\begin{array}{cc} S_{k} & ={\left(Y_{s,k}\right)}^{-1}, \end{array}$$

(52) $$\begin{array}{cc} P_{k} & =S_{k}S_{k}^{T}. \end{array}$$

Substituting $m_{k}$ and $P_{k}$ into (24)–(27), we can finally achieve the posterior intensity at time step *k*.

### 3.2. The State Extracting Method of the LSRCI-GM-PHD Approach

In this section, we present the state extracting method for our LSRCI-GM-PHD approach. As for our method, we use the preset threshold to prune the GM components with small weights. Then, we combine the GM components with the same label into one labeled GM component. Thus, these combined components can be applied for the next iteration. Since lots of GM components with small weights have been merged, the computational cost can be significantly reduced. At last, we extract the states and covariances of the multi extended target by combining the GM components with the same labels. Thus, the labeled states of multi extended targets can be achieved.

Let the updated GM component set be $G_{u}=\left\{l^{i},w_{i,k},m_{i,k},P_{i,k}\right\}$, where $i=1,2,\ldots ,N_{p}$, and $N_{u}$ is the number of GM components. Suppose $w_{pr}$ is the pruning threshold, we prune the GM components by

(53) $$G_{p}=\left\{\left(l^{i},w_{i,k},m_{i,k},P_{i,k}\right)|w_{i,k}>w_{pr}\right\}.$$

Using (53), we can remove the GM components with small weights from the updated GM component set. In fact, GM components with the same label have a high possibility to be associated with the same target. Let $\left\{w_{i,k}^{l},m_{i,k},P_{i,k}\right\}$ be the *i*-th GM component whose label is *l*. Then, we can combine these GM components by

(54) $$w_{l,k}=\sum_{i=1}^{n_{l}}w_{i,k}^{\left(l\right)},$$

where $n_{l}$ denotes the number of GM components with the label *l*.

We can merge the states and covariances of the GM components by

(55) $$m_{l,k}=\sum_{i=1}^{n_{l}}\frac{w_{i,k}^{\left(l\right)}}{w_{l,k}}m_{i,k}^{l},$$

and

(56) $$P_{l,k}=\sum_{i=1}^{n_{l}}\left(\frac{w_{l,k}^{\left(i\right)}}{w_{l,k}}P_{i,k}+\left(m_{i,k}^{l}-m_{l,k}\right){\left(m_{i,k}^{l}-m_{l,k}\right)}^{T}\right)$$

Using (54)–(56), the labeled states of multi extended targets can be extracted by

(57) $${\tilde{X}}_{k}=\left\{\left(l,m_{l,k},P_{l,k}\right)|w_{l,k}>w_{h}\right\},$$

where $w_{h}$ is the extract threshold. In this paper, we set $w_{h}=0.5$.

### 3.3. The Trajectory Constructing Method of the Proposed Approach

When the current estimated target set ${\tilde{X}}_{k}$ has been achieved in Section 3.2, it is very important to assign the estimated states with labels into their corresponding trajectories. Theoretically, a trajectory (representing one target) contains states with the same label. These states are ordered by different time steps. Due to the clutters and target detection loss, the estimated states and trajectory (belonging to the same target) may have different labels. Thus, the trajectory may be broken into several track segments. It may be very computational costly to manage these segments. In addition, for the sake of the target arising and disappearing, it is rather hard to confirm the beginning and end of the trajectory.

To solve the above problems, we, in this paper, propose our trajectory constructing method. As for our method, we divide the trajectories of the targets into three sets, named the current survival target set $C^{r}$, undetected target set $U^{d}$, and death target set $D^{e}$. Meanwhile, we also define the unconfirmed set $U^{c}$ to save the estimated states without associated trajectories. With these definitions, our method can solve the cases, such as the target birth, survival, death and, loss. The details of the presented method are given as follows.

(1) Assign the estimated states

As for our approach, states with the same label at different time steps are more likely to be generated by the same target. These states can be appended into one trajectory segment.

Let $u_{k-1}$ be the trajectory in $C_{k-1}^{r}\cup U_{k-1}^{c}$ at time step k−1; $l_{k-1}^{u}$ is the corresponding label of $u_{k-1}$, and $l_{k}^{x}$ is the label of the estimated state ${\tilde{x}}_{k}$.

With the above definitions, when $l_{k-1}^{u}=l_{k}^{x}$, we have

(58) $$\begin{array}{c} u_{k}=\left[u_{k-1},{\tilde{x}}_{k}\right], \end{array}$$

(59) $$\begin{array}{c} {\tilde{X}}_{k}={\tilde{X}}_{k}\setminus {\tilde{s}}_{k}, \end{array}$$

where ${\tilde{s}}_{k}=\left\{l_{k}^{x},{\tilde{x}}_{k},P_{k}\right\}$.

We can update $C_{k-1}^{r}$ and $U_{k-1}^{c}$ by

(60) $$\begin{array}{ccc} C_{k}^{r}=C_{k}^{r}\cup u_{k} & C_{k-1}^{r}=C_{k-1}^{r}\setminus u_{k-1}, & u_{k-1}\in C_{k-1}^{r}, \end{array}$$

(61) $$\begin{array}{ccc} U_{k}^{c}=U_{k}^{c}\cup u_{k} & U_{k-1}^{c}=U_{k-1}^{c}\setminus u_{k-1}, & u_{k-1}\in U_{k-1}^{c} \end{array}$$

When there are no labels equal to $l_{k}^{x}$ in $C_{k-1}^{r}\cup U_{k-1}^{c}$, we directly save ${\tilde{x}}_{k}$ into $U_{k}^{c}$.

Affected by clutters and target detection loss, several labels of the trajectories in $C_{k-1}^{r}\cup U_{k-1}^{c}$ may not appear in ${\tilde{X}}_{k}$. These trajectories can be considered the undetected targets at the current time step. Thus, we remove these trajectories from $C_{k-1}^{r}\cup U_{k-1}^{c}$, and add them into undetected set $U^{d}$

(2) Begin the trajectory

In practice, the estimated states are affected by targets and clutters. The associations between these discrete states are usually unknown beforehand. Therefore, it is rather hard to begin a trajectory. As for the RFS-based approaches, the clutters are assumed to follow the Poisson distribution. States (generated by clutters) with the same label have a rather low possibility to appear at two time steps consecutively. Thus, researchers commonly begin a trajectory when there are more than three associated states. In this paper, we follow this logic way to begin the trajectory.

Assume that *l* denotes the label of $u_{c,k}$, and *l* is not in sets $C^{r}$ and $U^{d}$. When $u_{c,k}$ satisfies the follow equation,

(62) $$length\left(u_{c,k}\right)\geq 3,u_{c,k}\in U_{c},$$

we can consider $u_{c,k}$ as a new trajectory segment. length(·) denotes the length of $u_{c,k}$. Then, we can remove $u_{c,k}$ from $U_{c}$, and implement the trajectory matching step.

(3) Matching the interrupted trajectories

Caused by clutter and missed target detection, trajectories belonging to the same target may have different labels. To join these trajectories into one trajectory, we match these trajectories by computing the statistic distance between them.

Let $u^{d}$ and $u^{c}$ be two trajectories of the same target. $u^{d}$ is the trajectory before interruption, and $u^{c}$ denotes the trajectory after interruption. Obviously, $u^{d}$ are in sets $U^{d}$. Suppose such a target misses detection at time step k−1, $x_{k-2}^{u}$ is the state of $u^{d}$ at time k−2, and $x_{k}^{c}$ is the state of $u^{c}$ at time step *k*. Here, we define the distance between $u^{d}$ and $u^{c}$ by

(63) $$d\left(x_{k|k-1}^{u},x_{k}^{c}\right)=\sqrt{{\left(x_{k|k-1}^{u}-x_{k}^{c}\right)}^{T}P_{u,k|k-1}^{-1}\left(x_{k|k-1}^{u}-x_{k}^{c}\right)},$$

where $P_{u,k|k-1}$ is the covariance of $x_{k|k-1}^{u}$. $x_{k|k-1}^{u}$, computed by the motion model (28), is the predicted state of $x_{k-2}^{u}$($x_{k-2}^{u}\to x_{k-1|k-2}^{u}\to x_{k|k-1}^{u}$).

We join $u^{d}$ and $u^{c}$ into one trajectory by

(64) $$c^{r}=\left\{\left[u^{d},x_{k-1|k-2},u^{c}\right]|d\left(x_{k|k-1}^{u},x_{k}^{c}\right)<T_{u}\right\},$$

where $T_{u}$ is the threshold, and $x_{k-1|k-2}$ is the predicted state of $x_{k-2}$.

When the distance between $u^{d}$ and $u^{c}$ is larger than the preset threshold, we consider these two trajectory segments as different targets and save $u^{c}$ into $C^{r}$.

For $u^{d}\in U^{d}$, if there do not exist states with the same label at two consecutive time steps, the trajectory $u^{d}$ can be considered as a death target. We remove it from the set $U^{d}$, and we put $u^{d}$ into the death set $D_{k}^{e}$.

The procedure of the presented trajectory constructing method can be seen in Table 1. We also summarize the proposed LSRCI-ET-GM-PHD approach in Table 2.

### 3.4. Computational Complexity

In this section, we analyze the computational complexity of our approach. In this paper, we focus on the improvement of the tracking performance of the conventional ET-GM-PHD approach. We directly adopt the same partitioning method of the ET-GM-PHD approach. Therefore, we only analyze the computational complexity of the updated density computation of the proposed approach. As for the conventional PHD approach without a gating method, the complexity of computing the updated density is nearly $O(N_{p}\cdot N_{W}\cdot N_{C})$. $N_{p}$ and $N_{W}$ are the number of possible partitions and cells, respectively. $N_{C}$ is the number of observations in the cell. For simplicity, we suppose each cell has the same number of observations. Assume that when using the gating method, the cell number is denoted by $N_{T}$. Obviously, $N_{T}\leq N_{W}$. Then, the complexity is nearly $O(N_{p}\cdot N_{T}\cdot N_{C})$. When the number of observations is large, the number of partition grows rapidly. The value $|N_{W}-N_{C}|$ would increase even more. Therefore, using the gating method, the computational complexity can be greatly reduced. In addition, we incorporate the SRCIF method into the ET-GM-PHD approach. The complexity of the SRCIF method is nearly $O(n_{d}^{3})$, where $n_{d}$ is the dimension of the state of the GM component. Thus, the total computational complexity of our approach in updated density computing is nearly $O(n_{d}^{3}\cdot N_{p}\cdot N_{T}\cdot N_{C})$.

## 4. Simulation Results

In this section, we validate the tracking performance of our LSRCI-ET-GM-PHD approach. A nonlinear scenario composed of five targets has been constructed in Section 4.1. Under such a scenario, we use the optimal sub-pattern assignment (OSPA) metric and RMSE to compare the tracking performance of the ET-GM-PHD [24], CK-EPHD [26], and our LSRCI-ET-GM-PHD approaches. Section 4.2 compares the simulation results of the three approaches under a certain number of clutters. Section 4.3 and Section 4.5 validate these approaches with various clutter ratios and detected probabilities, respectively.

### 4.1. Simulation Scenarios

In this section, we follow the way of [39] to construct the nonlinear scenarios. Let $x_{k}=\left[s_{k}^{x},v_{k}^{x},s_{k}^{y},v_{k}^{y},\alpha_{k}\right]$, where $\left(v_{k}^{x},v_{k}^{y}\right)$ is the velocity, $\left(s_{k}^{x},s_{k}^{y}\right)$ is the position, and $\alpha_{k}$ is the turn rate. With these definitions, we represent the nonlinear dynamic model by

(65) $$\begin{array}{cc} x_{k}= & \\ & \left[\begin{array}{ccccc} 1 & \frac{\sin(\alpha_{k-1}T)}{\alpha_{k-1}} & 0 & -\frac{1-\cos(\alpha_{k-1}T)}{\alpha_{k-1}} & 0\\ 0 & \cos(\alpha_{k-1}T) & 0 & -\sin(\alpha_{k-1}T) & 0\\ 0 & \frac{1-\cos(\alpha_{k-1}T)}{\alpha_{k-1}} & 1 & \frac{\sin(\alpha_{k-1}T)}{\alpha_{k-1}} & 0\\ 0 & \sin(\alpha_{k-1}T) & 0 & \cos(\alpha_{k-1}T) & 0\\ 0 & 0 & 0 & 0 & 1 \end{array}\right]x_{k-1}\\ & +\left[\begin{array}{ccc} \frac{T^{2}}{2} & 0 & 0\\ T & 0 & 0\\ 0 & \frac{T^{2}}{2} & 0\\ 0 & T & 0\\ 0 & 0 & 1 \end{array}\right]\varepsilon_{k-1}. \end{array}$$

Here, T=1 s is the sampling interval, and $\varepsilon_{k-1}$ is the noise. In this paper, $\varepsilon_{k-1}$ is defined by $\varepsilon_{k-1}∼N\left(\varepsilon_{k-1};0,Q\right)$. Q is the covariance matrix, denoted by $Q=diag(\sigma_{x,\varepsilon }^{2},\sigma_{y,\varepsilon }^{2},\sigma_{\alpha }^{2})$. $\sigma_{x,\varepsilon }$ and $\sigma_{y,\varepsilon }$ are the errors. In this paper, we set $\sigma_{x,\varepsilon }=\sigma_{y,\varepsilon }=1$ ${m/s}^{2}$, and $\sigma_{\alpha }=\pi /180$ rad. The initial states, appearing times, and disappearing times of five targets are listed in Table 3.

In addition, we depict the observation model by

(66) $$z_{k}=\left[\begin{array}{c} \arctan\left(\frac{s_{k}^{y}}{s_{k}^{x}}\right)\\ \sqrt{{\left(s_{k}^{x}\right)}^{2}+{\left(s_{k}^{y}\right)}^{2}} \end{array}\right]+\eta_{k}$$

where $\eta_{k}$, denoted by $\eta_{k}∼N\left(\eta_{k};0,R\right)$, is the observation noise. R is the covariance. In this paper, $\sigma_{\theta }=\pi /180$ rad and $\sigma_{r}=1$ m.

In multi extended target scenarios, the most widely used metrics are the OSPA distance and the generalized OSPA (GOSPA) distance [40]. The GOSPA requires the covariances for the estimated and true states. In this paper, we only estimate the states of multi extended targets. Thus, we adopt the OSPA distance as one of the metric. Let $X=\{x_{1},\ldots ,x_{m}\}$ and $Y=\{Y_{1},\ldots ,Y_{n}\}$ be two RFSs. *m* and *n* denote the numbers of elements in X and Y, respectively. $\Omega_{n}$ is the set of permutations of {1,2,…,m}, the second-order OSPA distance can be represented by

(67) $${\bar{d}}^{c}\left(X,Y\right)=\frac{1}{n}{\left(\min_{\varsigma \in \Omega_{n}}\sum_{i=1}^{m}d^{c}{\left(x_{i},y_{\varsigma (i)}\right)}^{2}+c^{2}\left(n-m\right)\right)}^{\frac{1}{2}}.$$

In (67), both X and Y are RFSs. $X=\{x_{1},\ldots ,x_{m}\}$ and $Y=\{Y_{1},\ldots ,Y_{n}\}$. *m* and *n* represent the number of elements in X and Y, respectively. $\Omega_{n}$ denotes the set of permutations of {1,2,…,m}. c>0 denotes the cut off factor, and $d^{c}\left(x,y\right)$ can be defined by

(68) $$d^{c}\left(x,y\right)=\min\left(c,d\left(x,y\right)\right).$$

In this paper, d(x,y) is the Euclidean distance. The cut off factor *c* is set to be 70.

**Figure 1:**
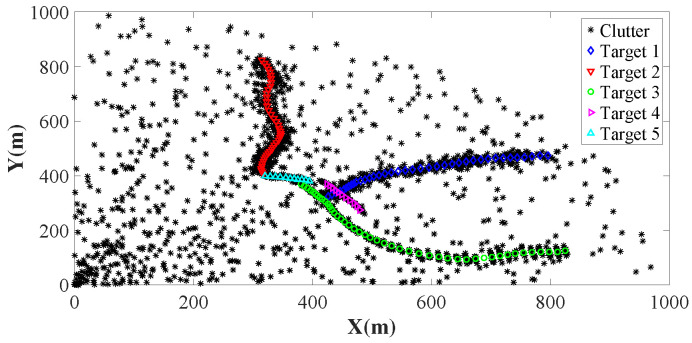
Ground-truth trajectories of five targets with the clutter number set to 20. The target trajectories are depicted by circle-solid lines with different colors, while the asterisks denote clutters.

For the parameters, we set the gating threshold $T_{u}=16$ in (64) (the same as [41]) and $T_{c}=0.5$. We also set the probability of survival as $p_{s}\left(x_{k}\right)=0.99$, and the probability of detection as $p_{d}\left(x_{k}\right)=0.95$, which are the same as [24]. The observations for each target follow the Poisson distribution. Here, we set the Poisson rate to be 20. In the detection region, the clutters are uniformly distributed. The angle and distance ranges of the clutters are (0,π/2) rad and (0,1000) m, respectively. The clutter in the observation set is generated by the uniform distribution. Here, the clutter ratio is 20. Using the above parameters, we demonstrate the trajectories of the five targets in Figure 1.

All of the simulation results are achieved using a computer with i7 processor, 8 GB RAM, and MATLAB 2016a.

### 4.2. Comparison of Estimation Accuracy on a Certain Clutter Ratio

**Figure 2:**
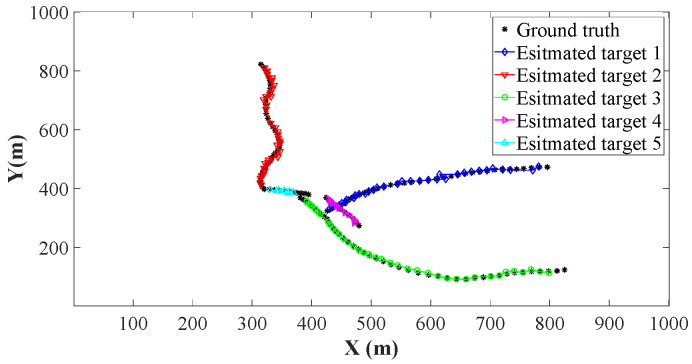
The estimation trajectories of the ET-GM-PHD approach. Here, we select black stars to represent trajectories of the ground truth. Meanwhile, the estimated trajectories of the five targets are denoted by curves with different colors and markers.

**Figure 3:**
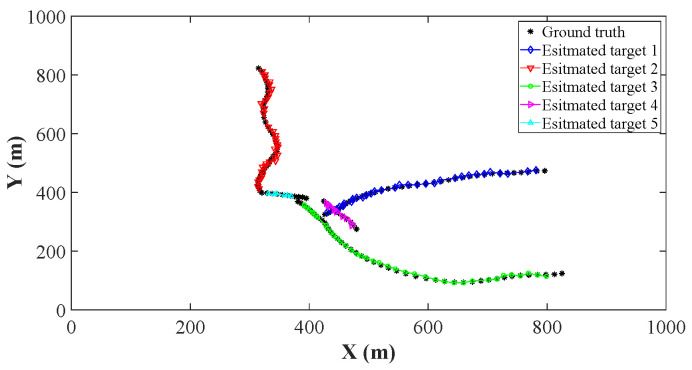
The estimation trajectories of the CK-EPHD approach. Here, we select black (dark) points to represent trajectories of the ground truth. Meanwhile, the estimated trajectories of the five targets are denoted by curves with different colors and markers.

**Figure 4:**
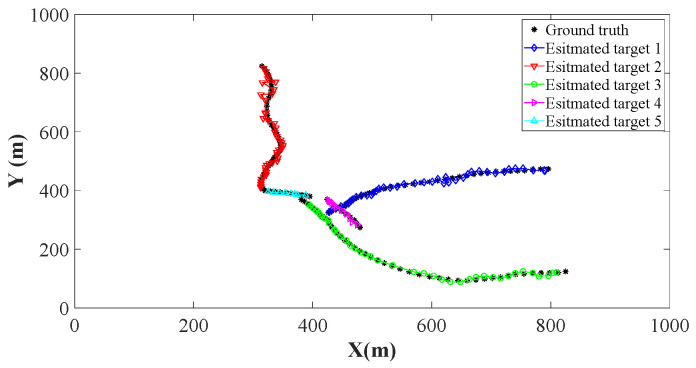
The estimation trajectories of the proposed LSRCI-ET-GM-PHD approach. Here, we select black (dark) points to represent trajectories of the ground truth. Meanwhile, the estimated trajectories of the five targets are denoted by curves with different colors and markers.

Using the parameters of Section 4.1, we implement the ET-GM-PHD, CK-EPHD, and our LSRCI-ET-GM-PHD approaches using a certain clutter ratio, where the clutter ratio is set to be 20. Each approach is performed with 500 Monte Carlo runs. For fairness, we use the labeled state extracting and trajectory constructing methods in Section 3.1 and Section 3.3 for all of the above approaches. Recall that the ET-GM-PHD approach is presented under the linear scenarios, and it cannot be directly applied in our nonlinear scenarios. The extended Kalman filter is one of the most widely used nonlinear target tracking approaches, which can be conveniently integrated into the ET-GM-PHD approach. We incorporate the extended Kalman filter into the ET-GM-PHD approach. Thus, it can be implemented in our scenarios. The estimated trajectories of these approaches are plotted in Figure 2, Figure 3 and Figure 4. From these figures, we can observe that the trajectories of estimated targets are demonstrated by the different color. Since all of the three approaches apply the trajectory constructing method of Section 3.3, the estimated states at different time steps can be well associated (assigned different colors in Figure 2, Figure 3 and Figure 4). We can also observe that several points of the ground truth have not been overlapped. That is to say, the target is missing at these time steps. Compared with Figure 2 and Figure 3, Figure 4 has the highest number of covered points. Benefiting from the significant performance of the SRCIF method in nonlinear target tracking, our LSRCI-ET-GM-PHD approach has the best estimation accuracy across all three approaches.

**Figure 5:**
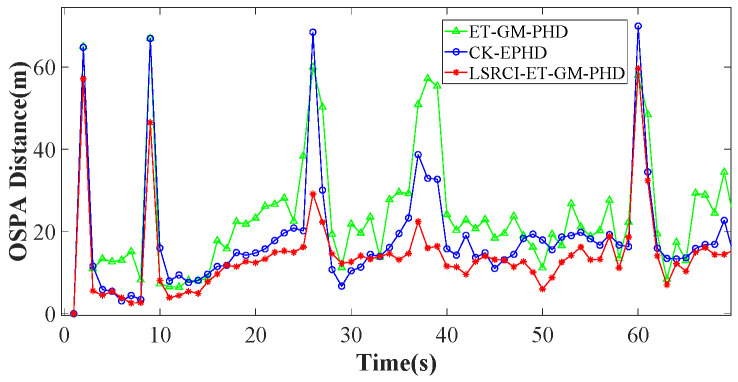
OSPA distances of the three approaches with the clutter ratio being 20.

Furthermore, we compare the OSPA distances of these approaches in Figure 5. In contrast to the ET-GM-PHD and CK-EPHD approaches, the curve of our LSRCI-ET-GM-PHD approach has smaller values. Notice that large OSPA distance denotes large tracking errors, the proposed LSRCI-ET-GM-PHD has the smallest tracking errors across all of these approaches.

**Figure 6:**
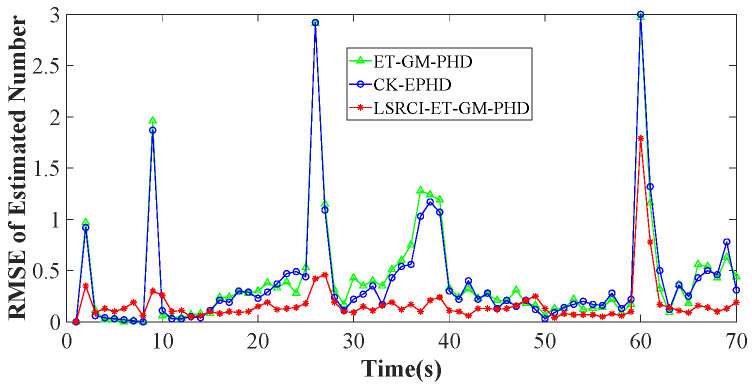
RMSEs of the three approaches with the clutter ratio being 20.

Furthermore, we plot RMSEs of the three approaches to estimate the number of targets in Figure 6. From Figure 6, we can observe that our LSRCI-ET-GM-PHD approach enjoys the smallest RMSE of the three approaches in Figure 6. In addition, we provide the numerical results of these approaches in Table 4. According to Table 4, the OSPA distance and RMSE of the LSRCI-ET-GM-PHD approach are the smallest in all of these approaches. Therefore, our LSRCI-ET-GM-PHD approach can achieve better accuracy in estimating states and the number of targets than the other two approaches.

### 4.3. Comparison of Estimation Accuracy on Various Clutter Ratios

**Figure 7:**
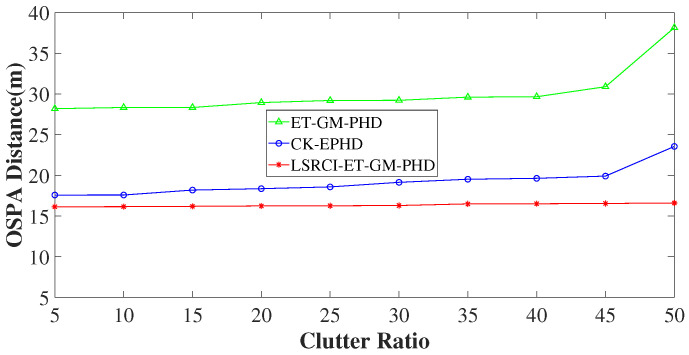
Averaged OSPA distances of the ET-GM-PHD, CK-EPHD, and our LSRCI-ET-GM-PHD approach along with the clutter ratio changing from 5 to 50.

**Figure 8:**
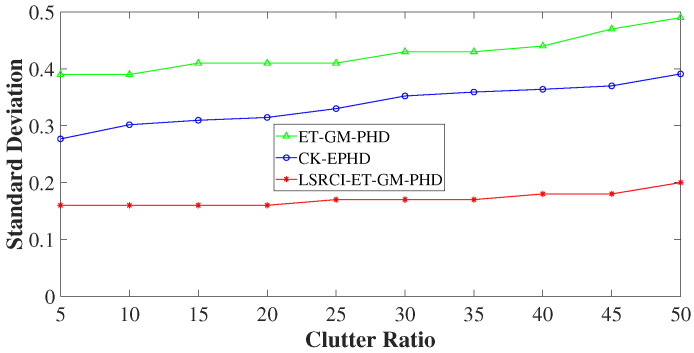
Averaged RMSEs of estimated number of the ET-GM-PHD, CK-EPHD, and our LSRCI-ET-GM-PHD approach along with the clutter ratio changing from 5 to 50.

For validating the effects of clutters on tracking accuracy, we change the clutter ratios and apply the three approaches. Here, the clutter ratio is varied from 5 to 50. The OSPA distances and RMSEs of these approaches under different clutter ratios are illustrated in Figure 7 and Figure 8. Since the ET-GM-PHD approach uses the the first-order Taylor series to approximate the nonlinear function of (29), it yields larger tracking errors than the other two approaches (seen in Figure 7 and Figure 8). From these figures, we can also see that the curves of the proposed LSRCI-ET-GM-PHD approach have the smallest values in all of the three approach. In addition, when the clutter ratio increases from 5 to 50, the OSPA distances and RMSEs of these approaches are enhanced. However, the changes of the proposed approach in Figure 7 and Figure 8 seem less than the other two. It is because of that we apply the SRCIF to estimate the means and covariances of the GM component, and we use the gating to decrease the effects of clutters. Thus, our LSRCI-ET-GM-PHD approach can achieve better tracking performance than the ET-GM-PHD and CK-EPHD approaches.

### 4.4. Comparison of Estimation Accuracy Under Different Detection Probabilities

In this section, we implement the ET-GM-PHD, CK-EPHD, and our LSRCI-ET-GM-PHD approach using different probabilities of detection. Here, the probability of detection is set to 0.85, 0.90, 0.95, and 0.99. The comparisons of these approaches are run with 500 Monte Carlo. The clutter number is set to be 20. Table 5 demonstrates the OSPA distances and RMSEs of the three approaches.

In Table 5, when the probability ranges from 0.85 to 0.95, the OSPA distances and RMSEs are rapidly decreased. Recall that when the probability of detection becomes high, the observation has a high probability of being generated by multi targets. Thus, the tracking errors can be reduced. When the probability of detection becomes 0.95 and 0.99, the OSPA distances and RMSEs are slowly decreased. The estimated results of the three approaches become stable. In addition, owing to the SRCIF and gating methods, our LSRCI-ET-GM-PHD approach has the least tracking errors in all of the three approaches, which yields the best tracking performance.

### 4.5. Comparison of the Estimation Accuracy Under Different Survival Probabilities

In this section, we change the survival probability from 0.80, to 0.85, 0.90, and 0.95. The clutter ratio and detection probability are the same as Section 4.2. All of the three approaches are implemented with 500 Monte Carlo. We list the results in Table 6.

Table 6 indicates that when $p_{s}$ changes from 0.80 to 0.95, the estimation accuracy of the three approaches increases. Compared with the ET-GM-PHD approach, the OSPA distances and RMSEs of the CK-EPHD and LSRCI-ET-GM-PHD approaches are smaller. Moreover, the OSPA distance and RMSE of our LSRCI-ET-GM-PHD approach changes more slowly than the other two. That means the estimation accuracy of our LSRCI-ET-GM-PHD approach is stabler than the other two approaches in the various survival probabilities.

## 5. Conclusions

In this paper, we have proposed the LSRCI-ET-GM-PHD approach. Such an approach can not only be employed to estimate the states and number of extended targets, but it can also be used to construct the trajectories of multi extended targets. Unlike the conventional ET-GM-PHD approach, we represent the densities of extended targets by a group of labeled GM components. These labeled GM components are predicted and updated under the SRCIF framework. Since the SRCIF is derived under the standard point target assumption, we came up with an observation extraction strategy to construct the candidate observation set. With such a strategy, only observations associated with the current states can be applied for the updating step of the SRCIF. Thus, computational costs can be saved. Meanwhile, we also present the state extracting method. With such a method, we can extract the states of multi extended targets from the updated GM components. In addition, a trajectory constructing method has been put forward. By constructing the associations between extracted states and trajectories, we can obtain steady trajectories of the multi extended targets. The simulation results show that our LSRCI-ET-GM-PHD approach outperforms the conventional ET-GM-PHD and CK-EPHD under the metrics of OSPA distance and RMSE.

This paper concentrates on improving the tracking performance of the conventional ET-GM-PHD approach in nonlinear multi extended target tracking. It simply adopt the existed partition method of the ET-GM-PHD approach. Other clustering methods for partition grouping may be integrated into our approach in future work. Otherwise, estimating the shapes of the extended targets by the ET-GM-PHD approach can be considered as another direction.

## Figures and Tables

**Table 1:** The trajectory constructing method.

– **Input:** Estimated state set ${\tilde{X}}_{k}$, survival target set $C_{k-1}^{r}$, undetected target set $U_{k-1}^{d}$, death target set $D_{k-1}^{e}$, and unconfirmed set $U_{k-1}^{c}$ – **Output:** Survival target set $C_{k}^{r}$, undetected target set $U_{k}^{d}$, death target set $D_{k}^{e}$, and unconfirmed set $U_{k}^{c}$ - Assign the estimated state set by (58) and (59). - Constructing the updated $C_{k}^{r}$ and $U_{k}^{c}$ by (60) and (61). - Add the left states of ${\tilde{X}}_{k}$ into $U_{k-1}^{c}$, $U_{k}^{c}=\left\{U_{k-1}^{c},{\tilde{X}}_{k}\right\}$. - Extract undetected trajectory segments, and achieve $U_{k}^{d}$ by adding them into $U_{k-1}^{d}$. - Begin a new trajectory $u_{c}$ by (62). - Compute the distance between $u_{c}$ and segments in $U_{k}^{d}$. - Find the interrupted trajectories, and joint them into one trajectory by (64). - Remove the interrupted trajectories from $U_{k}^{d}$, and add it into $C_{k}^{r}$. - Extract trajectories of death targets from $U_{k}^{d}$, and add it into $D_{k-1}^{e}$ to construct $D_{k}^{e}$. - Return $C_{k}^{r}$, $U_{k}^{d}$, $D_{k}^{e}$ and $U_{k}^{c}$.

**Table 2:** The LSRCI-ET-GM-PHD approach from time step k−1 to *k*.

– **Input:** Survival component set ${\left\{l_{i},x_{k-1}^{\left(i\right)},P_{k-1}^{\left(i\right)},w_{k-1}^{\left(i\right)}\right\}}_{i=1}^{L_{k-1}}$, and current observation set $Z_{k}$ – **Output:** Target number $N_{k}$, and estimated state set $X_{k}^{e}$ - Approximate the predicted means and square-root factor of covariances $\left\{m_{k|k-1}^{j},S_{k|k-1}^{j}\right\}$ of GM components by (34) and (35). - Substitute $\left\{m_{k|k-1}^{j},P_{k|k-1}^{j}\right\}$ into (19) to obtain the survival intensity $v_{s,k|k-1}\left(x\right)$, where $P_{k|k-1}^{j}=S_{k|k-1}^{j}{\left(S_{k|k-1}^{j}\right)}^{T}$. - Construct the birth intensity $\gamma_{k}\left(x\right)$, and obtain the predicted intensity $D_{k|k-1}\left(x\right)$ of (18) using $\gamma_{k}\left(x\right)$ and $v_{s,k|k-1}\left(x\right)$. - Cluster the current observation set $Z_{k}$ into different partitions by Table I in [24]. - Compute the candidate cell set ${\tilde{C}}_{k}$ of each partition by (44). - Evaluate the information forms $y_{k|k-1}^{j}$ and $Y_{s,k|k-1}$ corresponding to $m_{k|k-1}^{j}$ and $S_{k|k-1}^{j}$ by (46) and (47). - Obtain $y_{k}$ and $Y_{s,k}^{j}$ by solving (48) - Estimate $m_{k}^{j}$ and $P_{k}^{j}$ by $y_{k}^{j}$ and $Y_{s,k}^{j}$ using (50)–(52) - Prune the GM components of $D_{k|k}^{D}\left(x\right)$ using (53). - Achieve the estimated labeled state set $X_{k}^{e}$ by (57). - Construct the trajectory of the multi extended target by Table 1, and count the number of trajectory denoted by $N_{k}$. - Return $N_{k}$ and $X_{k}^{e}$.

**Table 3:** Initial states of targets.

Target	State	Appearing (s)	Disappearing (s)
1	[320,5,320,5,0]	1	40
2	[400,−5,400,5,0]	8	50
3	[375,5,375,−5,0]	25	70
4	[400,5,325,−5,0]	59	70
5	[325,−5,375,5,0]	59	70

**Table 4:** Averaged estimation errors.

Approach	OSPA (m)	RMSE
ET-GM-PHD	29.23	0.43
CK-PHD	17.29	0.25
LSRCI-ET-GM-PHD	16.19	0.17

**Table 5:** Tracking performance over different detection probabilities.

	$p_{d}=0.85$			$p_{d}=0.90$		
	**ET-GM-PHD**	**CK-EPHD**	**LSRCI-ET-GM-PHD**	**ET-GM-PHD**	**CK-EPHD**	**LSRCI-ET-GM-PHD**
OSPA (m)	35.80	21.09	18.49	29.56	18.77	16.54
RMSE	0.54	0.44	0.22	0.44	0.31	0.18
	$p_{d}=0.95$			$p_{d}=0.99$		
	**ET-GM-PHD**	**CK-EPHD**	**LSRCI-ET-GM-PHD**	**ET-GM-PHD**	**CK-EPHD**	**LSRCI-ET-GM-PHD**
OSPA (m)	28.25	17.13	15.93	28.13	16.59	15.84
RMSE	0.42	0.29	0.16	0.41	0.28	0.16

**Table 6:** Tracking performance over different survival probabilities.

	$p_{s}=0.80$			$p_{s}=0.85$		
	**ET-GM-PHD**	**CK-EPHD**	**LSRCI-ET-GM-PHD**	**ET-GM-PHD**	**CK-EPHD**	**LSRCI-ET-GM-PHD**
OSPA(m)	36.80	22.12	17.35	30.12	20.73	15.52
RMSE	0.55	0.39	0.22	0.48	0.32	0.17
	$p_{s}=0.90$			$p_{s}=0.95$		
	**ET-GM-PHD**	**CK-EPHD**	**LSRCI-ET-GM-PHD**	**ET-GM-PHD**	**CK-EPHD**	**LSRCI-ET-GM-PHD**
OSPA (m)	29.53	17.60	14.92	29.27	17.62	14.84
RMSE	0.43	0.27	0.15	0.42	0.27	0.14

## Data Availability

The original contributions presented in this study are included in the article. Further inquiries can be directed to the corresponding author.

## Abbreviations

The following abbreviations are used in this manuscript:

PHD	Probability hypothesis density
GM	Gaussian mixture
SRCIF	Square root cubature information filter
EPHD	Extended-target PHD filter
PMHT	Probabilistic multi-hypothesis tracker
VB	Variational Bayes
PMBM	Poisson multi-Bernoulli mixture
ET-GM-PHD	Extended target tracking using Gaussian mixture PHD
ART	Fuzzy adaptive resonance theory
